# Managing daily intensive care activities: An observational study concerning *ad hoc *decision making of charge nurses and intensivists

**DOI:** 10.1186/cc10341

**Published:** 2011-08-08

**Authors:** Heljä Lundgrén-Laine, Elina Kontio, Juha Perttilä, Heikki Korvenranta, Jari Forsström, Sanna Salanterä

**Affiliations:** 1 Department of Nursing Science, University of Turku, Lemminkäisenkatu 1, FI-20014 Turku, Finland; 2 Bureau of Administration, Turku University Hospital, PO Box 52, FI-20521 Turku, Finland; 3 Faculty of Telecommunication and E-Business, Turku University of Applied Sciences, Joukahaisenkatu 3 A, FI-20520 Turku, Finland; 4 Department of Anaesthesia and Intensive Care, Turku University Hospital, PO Box 52, FI-20521 Turku, Finland; 5 The Finnish Medical Association (FMA), PO Box 49, FI-00501 Helsinki, Finland

## Abstract

**Introduction:**

Management of daily activities in ICUs is challenging. ICU shift leaders, charge nurses and intensivists have to make several immediate *ad hoc *decisions to enable the fluent flow of ICU activities. Even though the management of ICU activities is quite well delineated by international consensus guidelines, we know only a little about the content of the real clinical decision making of ICU shift leaders.

**Methods:**

We conducted an observational study with the think-aloud technique to describe the *ad hoc *decision making of ICU shift leaders. The study was performed in two university-affiliated hospital ICUs. Twelve charge nurses and eight intensivists were recruited. Observations were recorded and transcribed for qualitative content analysis using the protocol analysis method. The software program NVivo 7 was used to manage the data. The interrater agreement was assessed with percentages and by Cohen's κ.

**Results:**

We identified 463 *ad hoc *decisions made by the charge nurses and 444 made by the intensivists. During our data collection time, this breaks down to over 230 immediately made decisions per day (24 hours). We divided the *ad hoc *decision making of ICU shift leaders into two types: process-focused and situation-focused. Process-focused decision making included more permanent information, such as human resources, know-how and material resources, whereas situation-focused decision making included decisions about single events, such as patient admission. We named eight different categories for ICU *ad hoc *decision making: (1) adverse events, (2) diagnostics, (3) human resources and know-how, (4) material resources, (5) patient admission, (6) patient discharge, (7) patient information and vital signs and (8) special treatments.

**Conclusions:**

ICU shift leaders make a great number of complex *ad hoc *decisions throughout the day. Often this decision making involves both intensivists and charge nurses. It forms a bundle that requires versatile, immediate information for a successful outcome. In the future, we need to investigate which information is crucial for *ad hoc *decision making. These challenges should also be emphasised when information technology programs for ICU care management are developed.

## Introduction

Many and varied multiprofessional decisions are made when running daily ICU activities. These result in both clinical and managerial orders to support patient care and ICU work flow. Decision making related to immediate care must often be made quickly in response to the changing condition of the patient. This decision making is usually supported by patient information systems that are designed to provide detailed individual, patient-focused information [1]. However, decision making concerning direct patient care is difficult or even impossible to complete if the managerial decisions are made inaccurately and/or with a delay.

Within fast-paced ICU environments, many of these managerial decisions have to be made immediately, *ad hoc*. By '*ad hoc *decision making', we mean critical judgements that are needed for a specific purpose at a precise moment with the goal of ensuring instant and adequate patient care and a fluent flow of activities in the ICU. Shift leaders, intensivists and charge nurses in ICUs are responsible for coordinating, planning and assessing the daily activities of the units and are those who usually make these managerial *ad hoc *decisions. ICU shift leaders' duties and tasks are a mixture of direct patient care, supervision of care and administrative work. This is why many decisions have to be considered both from the clinical and the managerial points of view.

The decision making of ICU professionals has been the focus of several studies (see, for example, [2-5]). In addition, the need for teamwork, verbal communication and information has been found to be crucial for task coordination and work performance in ICUs [6-8]. However, the focus of previous studies has been mainly on the individual level or on direct patient care.

In recent years, precise guidelines and best practice instructions have been developed to guide the work flow of ICUs and managerial decision making. Despite these efforts, the *ad hoc *decision making of ICU shift leaders is poorly supported by information systems. It is common for information needed for decision making to be gathered into different information systems and other platforms. In addition, part of the decision making is based solely on the memory and work experience of the ICU shift leader. With a growing information load, it is essential that precise, consistent and timely information be available for accurate and correct decision making exactly when it is needed [9,10]. However, we do not have a clear picture of what decisions the ICU shift leaders make.

In this study, we focused on the unit-level decisions which are made *ad hoc *by ICU shift leaders to ensure the fluent flow of activities. The specific purpose of the study was to identify the *ad hoc *decisions of ICU shift leaders by evaluating their cognitive processes during their daily work.

## Materials and methods

### Context and setting

We conducted this study in two university hospital ICUs in Finland from 25 April to 23 June 2007. Both of the ICUs are mixed medical-surgical units with 24 (ICU1) and 22 (ICU2) beds Both units are run by full-time intensivists with 24-hour coverage. Together the units take care of over 4,000 patients annually. In-house intensivists and qualified charge nurses manage the daily activities of the units in both ICUs. The ICUs are closed-model units in which patients are admitted by the ICU physician who specialises in surgery, anaesthesia or internal disease. The charge nurses of the units are registered nurses experienced in directing nursing staff. Decisions related to direct patient care are made jointly by the specialist consultants and the ICU team. Decisions concerning ICU work flow are made within the ICU team. Both of the study units use Centricity™ Critical Care [11] information systems for electronic patient documentation. More detailed characteristics of the ICUs are given in the Table 1.

**Table 1:** Characteristics of the participating ICUs

Characteristics, 2007	ICU 1	ICU 2
Patients, *n*	1,727	2,615
Surgery	71.4%	56.3%
Conservative treatment	28.6%	43.7%
Mean LOS^a^, days	3.4	2.0
Mortality	7.8%	3.8%
Fellows, *n*	4	4
Residents, *n*	1	1 or 2
Assistant physicians, *n*	3	N/A
Head nurses, *n*	2	1
Staff nurses, *n*	3	2
Registered nurses, *n*	112	69
Practical nurses, *n*	9	12

^a^LOS = length of stay; N/A = not applicable.

### Study design

We performed an observational study using the think-aloud technique in a real clinical setting. The basis of the think-aloud technique is to ask participants to verbalise their performance while performing their tasks and duties. Researchers have found this technique to be an appropriate method for investigating and revealing complex cognitive processes in a real-life context [12,13]. Our purposeful sample consisted of 12 charge nurses and 8 intensivists equally at both hospitals. We conducted the collection of data during different shifts: 15 morning shifts, 4 evening shifts and 1 night shift. Data collection was also performed on different days of the week, including three weekend shifts. The data collector followed each participant individually from the beginning of the eight-hour work shift. The role of the data collector was to remind participants to talk aloud during the observations. All sessions were recorded using an MP3 player. One researcher (HLL), who was familiar with intensive care, conducted all the sessions.

### Data analysis

The data were typed out into a .txt file in an authentic form. Since the recorded data included each participant's speech during his or her shift, irrelevant parts that were not related to the focus of our study's purpose were omitted. Before the analyses, the text files were read several times and situation-related notes were made. These situation-related notes were made to detect which decision was related to which situation at the time of observations (for example, staff ratios to isolation). This supported the final analyses and understanding of the rapidly changing situations and ICU-specific concepts related to the complex thinking of the study participants. Qualitative content analysis was then used to identify each decision made by the participants. Qualitative content analysis is 'a research method for subjective interpretation of the content of text data through a systematic classification process of coding and identifying themes or patterns' [14] (p. 1278). Each recording was analysed separately with the aim of detecting the decisions of both professional groups.

Our analysis consisted of three phases: free-model coding, tree-model coding and application of the protocol analysis. A computer-assisted qualitative software program, NVivo 7 (QSR International, Doncaster, Victoria, Australia) [15], was used to support the management of the coding in our analysis. First, the free-model coding was used to identify decisions made by the participants. This meant that all utterances which included decision making were coded. Then we continued to organize the decisions hierarchically into clusters (tree-model coding). Some of the hierarchical single codings were still in several categories during this phase.

In the next phase, the hierarchical categories were compared and those with close contents were integrated, such as human resources and the know-how of personnel. All the coding phases were first performed by one researcher (HLL). After this, two researchers (HLL and EK) independently analysed all of the precoded data. We ensured the reliability with intercoder agreement percentages and the consistency of coding with Cohen's κ values (Table 2).

**Table 2:** Categories, amounts, coding frequencies of *ad hoc *decisions, number of *ad hoc *decisions, interrater reliability values and observation times

Categories of *ad hoc *decisions	Shift leaders	Intensivists (*n *= 8)	Charge nurses (*n *= 12)
Process-focused, *n *(%)			
1. Human resources and know-how	291 (32.1%)^b^		291 (63%)^b^
2. Material resources	22 (2.4%)		22 (4.8%)
Situation-focused, *n *(%)			
3. Patient admissions	32 (3.5%)	3 (0.7%)	29 (6%)
4. Patient information and vital signs	246 (27%)^b^	174 (39%)^b^	72 (16%)^b^
5. Special treatments	171 (19%)^b^	147 (33%)^b^	24 (5%)
6. Diagnostics	86 (9.5%)	86 (19%)^b^	
7. Adverse events	1 (0.1%)		
8. Patient discharges	58 (6.4%)	34 (8%)	24 (5%)
IRR^a ^(%)/Cohen's κ		97.0/0.92 to 1.0	91.0/0.90 to 1.0
Total *ad hoc *decisions, *n*	907	444	463
Total observation time, hours	92	30	62

^a^IRR = interrater reliability; ^b^largest categories.

Finally, we applied protocol analysis and analysed the levels of verbalization in sentences. This was done to identify *ad hoc *decisions, that is immediately made decisions. In the protocol analysis, the verbalization of study subjects was classified into three levels. These levels were used to describe the reproduction of information from the memory of a study subject while he or she was performing a task. First- and second-level verbalizations were considered reliable because these levels are assumed to reveal the content of the short-term working memory [12]. By applying protocol analysis, we were able to code each decision into a different level. First- and second-level decisions were considered *ad hoc *decisions. Verbalizations of decisions at levels 1 and 2 were clearly and immediately expressed as well as clearly targeted. These decisions needed to be made rapidly, and the decision maker sought either an immediate solution or more information to be able to conclude his or her decision without delay. For the third-level decisions, participants needed additional information and the solutions required for decision making were not immediately essential. The third-level decisions were not *ad hoc *decisions and included those related to a patient discharge plan, such as, 'We are going to extubate probably tomorrow, and if all goes okay he will be discharged'.

A study on protocol analysis by Ericsson and Simon [12] and an article by Lundgrén-Laine and Salanterä [16] discussed the methodological bases of protocol analysis and the levels of the analysis in more detail.

### Ethical issues and participants

The study was approved by the hospital districts' authorities. In our study, we followed the Finnish national legislation and ethical principles [17]. No data are personally identifiable. The inclusion criteria for the study subjects were that the participant had to (1) be able to voluntarily, capably and competently talk aloud during work situations and (2) have current ICU shift leadership experience. A written consent form was obtained from each participant, and each received written and oral instructions before the observations.

## Results

Four of the intensivists were men and four were women, with their ICU work experience ranging from 4 to 22 years. Three of the twelve charge nurses were men, and nine were women. Their ICU work experience ranged from 5 to 32 years. None of the participants withdrew from our study during the observations.

The recordings varied from two to six hours per informant (mean duration 4 hours, 40 minutes), and the length of the recordings depended on the intensity of the shift. Altogether we recorded 92 hours of data. In our analysis, we identified 444 *ad hoc *decisions made by the intensivists and 463 *ad hoc *decisions made by the charge nurses. This breaks down to nearly 10 *ad hoc *decisions per hour or 240 *ad hoc *decisions per day made by the ICU shift leaders. If we consider the number of decisions per decision maker, these data break down to almost 56 *ad hoc *decisions per intensivist and 39 decisions per charge nurse.

The *ad hoc *decision making of intensivists and charge nurses covered the entire patient care process from admission to discharge. On the basis of our analysis, we identified eight decision-making categories: (1) adverse events, (2) diagnostics, (3) human resources and know-how, (4) material resources, (5) patient admission, (6) patient discharge, (7) patient information and vital signs and (8) special treatments.

**Figure 1:**
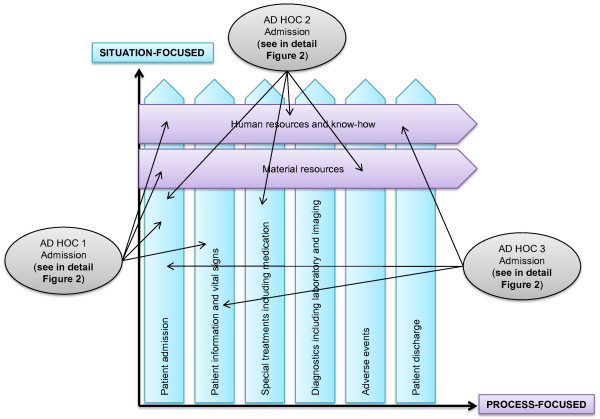
**Process-focused (horizontal) and situation-focused (vertical) *ad hoc *decision making**.

These categories were in turn divided into two different types of decision making: process-focused and situation-focused (Figure 1). In our data, the process-focused decision making represented *ad hoc *decision making that was related to permanent events. In addition, the process-focused *ad hoc *decisions were made concerning the entire unit, and they affected the work flow of the whole ICU. Human resources, know-how and material resources represent process-focused decision making (Figure 1, horizontal type).

The situation-focused *ad hoc *decision making concerned incidents that occurred at a certain moment. Typically they were single incidents or concerned one patient or individual, such as patient admission, adverse events, diagnostics, patient information and vital signs, special treatments and patient discharge (Figure 1, vertical type).

Most *ad hoc *decisions belonged to the categories of human resources and know-how (32.1%), patient information and vital signs (27%) and special treatments (19%), representing both process-focused and situation-focused decision making. These categories were followed by diagnostics (9.5%) and patient discharge (6.4%). The fewest *ad hoc *decisions made were related to patient admission (3.5%), material resources (2.4%) and adverse events (0.1%).

Table 2 summarises the named *ad hoc *decision-making categories, the frequencies and percentages of our coding, the participants in the study, the total observation times and intercoder reliability values with Cohen's κ values. In Table 3, we present the definitions for the named *ad hoc *decision-making categories.

**Table 3:** Definitions of *ad hoc *decision-making categories and examples

*Ad hoc *category	Definition	Examples
1. Adverse events	An injury related to medical management	'Concerning this pinprick accident, I have to order a blood test for her immediately'.
2. Diagnostics	Patient diagnosis, including laboratory and radiology results affecting ICU work organization	'Today's X-ray is OK, so she will be ready to leave'.
3. Human resources and know-how	The amount of staff resources, the knowledge of the ICU personnel and follow-up information and reports	(1) 'In that room, there is one very experienced nurse. It is enough. I'll keep it that way'.
		(2) ‘Two RNs are needed for this patient in the following shift’.
		(3) ‘RN XX will be named in the trauma team in the following shift’.
4. Material resources	Materials needed for ICU patient care	‘This isolation room is reserved for this patient in the next shift’.
5. Patient admission	Acceptance of patients for ICU care	‘The recovery room is booked up, so we have to take this patient immediately’.
6. Patient discharge	Acceptance of patient transfer from ICU care	‘If the bleeding stops, he will be discharged when a new patient is introduced’.
7. Patient information and vital signs	Intensive care-specific patient information and patient's intensive care- specific condition	(1) ‘We will put her to sleep and then deal with the AF’.^a^
		(2) ‘The patient with the highest nursing intensity will need two nurses in the evening shift’.
		(3) The patient’s invasive blood pressure values are too low, and he is not ready for discharge’.
8. Special treatments	ICU-specific care and medication administration	(1) ‘We will make the sterna closure tomorrow, so cardiologist consultation today’.
		(2) ‘Our unit is ready to admit a patient who needs haemodialysis’.
		(3) ‘With this patient, we will start an inotrope infusion and ask for a cardiology consultation’.

^a^AF = atrial fibrillation.

### Human resources and know-how

Most of the *ad hoc *decisions made by ICU shift leaders were about human resources and know-how (32.1%). The main objective of this decision making was to manage the number of ICU personnel, to ensure sufficient and appropriate resources or to compensate for the know-how levels needed so that patient care could be ensured in all situations around the clock. Remarkably, all the *ad hoc *decisions in this category were made by the charge nurses; however, they typically made these decisions in coordination with other ICU colleagues or negotiated in multidisciplinary teams before personally making judgments. In addition, many of the *ad hoc *decisions identified in this study dealt with a different kind of follow-up data needed for statistics or research. Typical *ad hoc *decisions under this category are presented in Table 3.

### Patient information and vital signs

The second most frequent *ad hoc *decisions were made concerning patient information and vital signs (27%). This category covered very intensive care, specific patient information and the present condition of a patient fulfilling the criteria for essential ICU care. These *ad hoc *decisions dealt, for example, with issues related to the mode of isolation, patient care intensity, critical problems regarding vital functions or changes required immediately for the intensive care plan. The *ad hoc *decision making in this category was also closely connected to other *ad hoc *decisions, such as those concerning admissions or discharges. For example, high patient care intensity led to the next *ad hoc *decision: which of the patients would stay and who would be moved to another ward. About 40% of these decisions were made by the intensivists, and these decisions comprised most of the *ad hoc *decisions they made (Table 2). The *ad hoc *decisions under this category are presented in Table 3.

### Special treatments

*Ad hoc *decision making related to special treatments (19%) was the third-largest category. Under this category, we named all those *ad hoc *decisions which dealt with ICU-specific care and administration of medication. ICU-specific care covered the treatments which were possible to perform only under intensive care circumstances, such as hyperbaric oxygen therapy, care of patients with intra-aortic balloon pumps or treatments performed by specialised consultants. Under this category, we also named administration of ICU-specific medication, such as medication that requires intensive monitoring (for example inotropes, vasodilators or sedatives). Examples of these *ad hoc *decisions are presented in Table 3.

### Diagnostics and patient discharge

Part of the *ad hoc *decision making of ICU shift leaders was related to diagnostics and patient discharge (9.5% and 6.4%, respectively). Only the intensivists made *ad hoc *decisions about diagnostics, covering one-fifth of all *ad hoc *decisions they made. An example of an *ad hoc *decision related to diagnostics is, 'Diagnosis confirmed today induces immediate changes in the patient's care plan'. The *ad hoc *decisions concerning patient discharge, such as, 'This patient is ready for discharge', were made by both the intensivists and the charge nurses (see also Table 3).

### Patient admission, material resources and adverse events

*Ad hoc *decisions about patient admission, material resources and adverse events constituted only a small portion of decisions made by the shift leaders. Intensivists and charge nurses made decisions about patient admission. Examples of these *ad hoc *decisions are 'Four beds should be booked for elective patients during the present shift' or 'The patient from the ER should be admitted immediately'. Decisions about material resources and adverse events were all identified from the data sets of the charge nurses. Examples of these are 'A special mattress is needed for the incoming patient' or 'The pinprick accident should be managed immediately according to the treatment protocol' (see also Table 3).

### A bundle of *ad hoc *decisions

**Figure 2:**
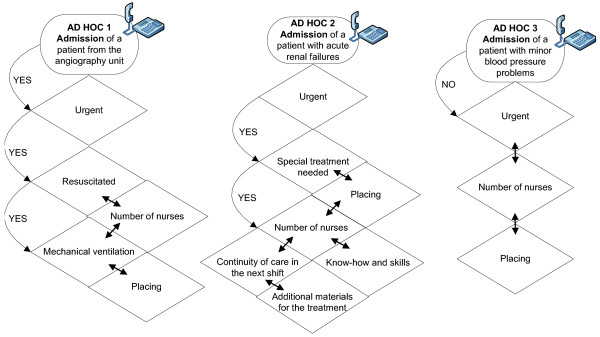
**Examples of *ad hoc *decision-making bundles**.

Most of the *ad hoc *decisions made by the ICU shift leaders were not isolated decisions but event-based, complex combinations of several decisions made by both the intensivist and the charge nurse. In addition, the *ad hoc *decision making of ICU shift leaders did not follow a linear process, and it varied between decision makers as well as in changing situations. The *ad hoc *decision making was a multidisciplinary process in which the roles of ICU shift leaders were not so well-defined. One *ad hoc *decision made by either of the shift leaders created a bundle of *ad hoc *decisions that were needed to complete the task. Typically, both process-focused and situation-focused *ad hoc *decision making was then needed. Figure 1 and *ad hoc *decisions 1, 2 and 3, explained in detail in Figure 2, show some simplified examples of the *ad hoc *decision-making bundles during the daily management of ICU activities. These examples have been extracted from our data. For example, different kinds of *ad hoc *decisions have to be made each time a new patient is admitted to the ICU, depending on various factors such as patient condition, urgency level, staffing and patient load.

## Discussion

To our knowledge, our study is the first to describe the *ad hoc *decision making of ICU shift leaders, including intensivists and charge nurses, during the management of daily ICU activities. Our study shows that ICU shift leaders make numerous *ad hoc *decisions when managing the daily activities of their units. Our analysis revealed that almost 10 immediately needed *ad hoc *decisions per hour are made by the shift leaders, which presents great challenges for immediate information retrieval.

We identified eight *ad hoc *decision-making categories for ICU shift leaders: (1) adverse events, (2) diagnostics, (3) human resources and know-how, (4) material resources, (5) patient admission, (6) patient discharge, (7) patient information and vital signs and (8) special treatments. These categories covered the whole ICU care process from patient admission to patient discharge. Even if the *ad hoc *decisions of the shift leaders differed, the goals and the content underlying their decisions were parallel. For example, intensivists made *ad hoc *decisions about incoming patients and who would be admitted first (patient admission). At the same time, charge nurses, for their part, made *ad hoc *decisions about the timing and placement of incoming patients (patient admission).

Most of the identified *ad hoc *decisions of the ICU shift leaders were connected to intensive care-specific patient information and the condition of the patient, human resources and know-how of the personnel and special treatments performed in intensive care settings (nearly 80%). This result confirms the basic role of ICU work, in which the most critically ill patients are taken care of by multiprofessional teams and the most demanding treatments can be performed by an adequate number of team members and specialised staff. In addition, our study results suggest that the *ad hoc *decision making of ICU shift leaders is complex and overlapping. These findings are supported by previous research on the decision making of ICU personnel [18,19].

Most *ad hoc *decision making concerns human resources and know-how. Similar results have been found in previous studies [7,20] in which the information requirements of the ICU patient care team and multidisciplinary care team in an emergency department have been investigated. Results of previous studies have shown that many clinical questions contain organizational elements which are essential for the coordination of the work.

Both our study and those of Reddy *et al*. [7,20] showed that clinical decision making is an important part of *ad hoc *decision making, and it is often connected with organizational issues. In our study, the clinical *ad hoc *decisions were mainly made by the intensivists, followed by several *ad hoc *decisions related to organizational issues made by charge nurses. In Reddy and Spence's study [7], for example, the patients' conditions were evaluated in relation to the need for beds. Similar aspects were found in our data. An *ad hoc *decision made by the intensivist regarding special treatment created several *ad hoc *decisions related to organizational issues such as placement of the patient, the availability and capability of the nurse, the timing of the treatment and ensuring the continuum of care from shift to shift.

In our study the charge nurses made most of the organizational decisions, which mainly concerned human resources and know-how. These organizational elements seem to contribute significantly to the work flow of the ICU. Our results might reflect the organizational culture of the ICUs investigated and how the work was organised and shared by the ICU team. It would be interesting to study this issue further in other ICUs at both the national and international levels.

The amount of different *ad hoc *decisions gives us information about which *ad hoc *decisions dominate the ICU shift leaders' work. However, it does not reveal the importance of the *ad hoc *decisions made. In our analysis, we found that the fewest *ad hoc *decisions made were related to diagnostics, patient discharge, patient admission, material resources and adverse events. However, all of these include extremely important decisions which affect the outcomes of ICU patient care.

Especially in urgent situations, when, for example, patient admission and discharge decisions have to be made immediately, all decisions should be based on accurate, clearly defined information. In previous studies, delays in patient admissions or after-hours discharges, for example, have been found to be associated with patient mortality [21,22]. Diagnostics play an important role in patient care in the ICU, and this category is closely connected to both admission and discharge decisions as well as to immediate changes that are required in care plans. *Ad hoc *decision making related to adverse events is often vital, since poorly thought out decisions can cause serious complications and even patient mortality [23,24].

ICU care management, coordination and decision-making procedures are strongly supported by the international consensus guidelines [25-27]. These guidelines contain definitions and objectives for the quality of ICU performance. In addition, recommendations for optimal ICU care are provided. However, these guidelines have mainly focused on the material resources needed at certain ICU care levels or in direct patient care, as well as on vital problems of ICU patients. Process-focused decision making has received little attention, and improvements in process performance from the multiprofessional point of view are needed [28,29].

Our results reveal that studies concerning only one professional group or one decision-making area are not enough when complex and fast-paced environments such as ICUs are investigated. In our study only very few ad hoc decisions were made by one person. Instead they were a chain of decisions made by several persons. The *ad hoc *decision making of ICU shift leaders is a team process whereby the final outcome is achieved with the collaboration of charge nurses and intensivists, which requires that several *ad hoc *decisions be made.

Our study has some strengths and some limitations. One limitation of our study is that we conducted it in only two ICUs and in one country. However, within these two 'full-service' ICUs, both of which have large ICU patient populations, we managed to obtain rich and saturated data. Our study ICUs were not randomly selected; rather, their selection was purposeful and based on the facts that both units take care of patients with various medical and surgical problems and both have similar operation models in their care organization. A purposeful sample also offered data of good quality. In addition, we did not evaluate the diurnal variation in decision-making frequency, which would be interesting to assess in the future. We hypothesized that more ICU daily activities are performed during morning shifts and created more stress on those choosing the 15 morning shifts to be observed. However, we suggest that the variation in decision-making frequency is more dependent on clinical situations than on working shifts. Our study does not provide information about the quality of shift leaders' decisions (that is, good and bad decisions). To identify the quality of the *ad hoc *decisions made, the whole decision-making process should be followed until the end and the final outcomes related to each decision should then be evaluated. Because of the type of data we collected, we cannot generalise our results *per se*, but other researchers can utilise the results in future research by testing them in other ICU settings.

A challenge in our study was combining the think-aloud technique with protocol analysis, especially the difficulty of compiling research on decision making in real settings. Think-aloud is used quite often in observational studies in health care [4,30,31]. However, protocol analysis in combination with think-aloud has rarely been used in acute clinical settings in health care. Alternatively, we could have used pure observation or introspection as a method of investigating the *ad hoc *decision making of shift leaders. However, with observation, a researcher might miss or misread a significant part of decision making. On the other hand, introspection involves interpretations by the participants themselves, which might affect the results (see, for example, [32]). In our study, we were interested in evaluating *ad hoc *decisions that had to be made immediately. It is not likely that the Hawthorne effect would play a significant role in this kind of decision-making situation. In our study, the think-aloud technique combined with protocol analysis functioned well. The participants in our study were capable of verbalizing their thinking. In addition, with protocol analysis, we were able to find and classify the *ad hoc *decision making.

The observational study design itself is a big challenge when complex phenomena are investigated in ICU settings. Even the shadowing of one participant whose work is to make many different decisions all the time was demanding. In our study, we used only one observer and recorder with one participant during one shift with the aim of covering the whole ICU situation as well as possible. Many details should be considered carefully when more than one decision maker, or shared decision making, is observed simultaneously. These details are, for example, time labels in starting and ending multiple decisions and differentiating *ad hoc *decisions aimed at the same targets from other decisions made at the same time. Combining handheld computers with work observations as Westbrook and Ampt tested in their study [33] using our study method could be an entirely novel way to look at this phenomenon in the future.

In the future, the reliability and generalization of our results should be validated by national and international multicentre studies in which the information variables that are crucial for *ad hoc *decision making in the ICU. The international perspective is supported by the facts that the basic targets of ICU care are internationally quite similar and the decision making of ICU personnel has also been found to be internationally comparable [34]. In addition, the processes of ICUs are internationally quite well defined and guided by international consensus guidelines supporting the managerial decision making of ICU professionals and standardizing the policies of the ICUs. However, different cultures and team functions might have some effect on decision-making processes in different ICUs.

## Conclusions

Our study describes the *ad hoc *decision making of ICU charge nurses and intensivists during the daily management of ICU activities. This study highlights the complex and fundamental phenomenon of *ad hoc *decision making in a real clinical ICU context. We have shown that ICU shift leaders make a vast number of *ad hoc *decisions concerning the entire ICU care process. Organizational issues are greatly emphasised. In addition, the *ad hoc *decision making of ICU shift leaders appears to be a multiprofessional process in which several *ad hoc *decisions are needed to complete one task. By identifying ICU shift leaders' *ad hoc *decisions and this multiprofessional process, we will be able to reveal what kind of information is needed in challenging clinical settings. Further research is required to identify what information needs are fundamental for ICU shift leaders in *ad hoc *decision-making situations. In the future, this will help us to develop electronic decision-making support and management systems for ICUs.

## Key messages

• ICU shift leaders make numerous immediate decisions during the management of daily activities.

• The *ad hoc *decision making during the management of daily ICU activities is a multiprofessional process, and the final outcome is usually achieved through multiple immediate decisions, a bundle of *ad hoc *decisions.

• The *ad hoc *decision making of ICU shift leaders is not a linear process, and it involves various areas of ICU care. The organizational issues are highly emphasised throughout the daily management of ICU activities.

• In the future, the *ad hoc *decision making of ICU shift leaders and access to the most crucial information should be confirmed.

## Competing interests

The authors declare that they have no competing interests.

## Authors' contributions

HLL designed the study, performed the observations, analysed the data and drafted the manuscript. EK acted as an intercoder and made comments on the manuscript. HK, JP and JF helped to draft the manuscript. SS supervised the study, assisted in the design of the study and prepared the final manuscript with HLL. All authors read and approved the final manuscript.
